# Epidemiological characteristics of respiratory diseases in emergency department patients from different ethnic groups in the Atushi region, Xinjiang

**DOI:** 10.3389/fmed.2025.1569629

**Published:** 2025-06-16

**Authors:** Jin Ma, Xiaolong Zhu, Qi Tang, Shifang Liu, Weiwei Zhou

**Affiliations:** ^1^Department of Emergency Medicine, the People’s Hospital of Atushi, Atushi, China; ^2^Administration Office, the People’s Hospital of Atushi, Atushi, China

**Keywords:** epidemiological, emergency department, ethnic, respiratory diseases, region’s climate

## Abstract

The Xinjiang region of China has long been a high-prevalence area for respiratory diseases, especially pulmonary tuberculosis and chronic obstructive pulmonary disease (COPD). This retrospective, cross-sectional study aimed to analyze the epidemiological characteristics of patients visiting the emergency department (ED) of the People’s Hospital of Atushi in Xinjiang, China, from January to December 2024. This study focused on the prevalence and distribution of respiratory diseases. A total of 8,837 patients were included, and respiratory diseases emerged as the most prevalent condition, accounting for 35.2% of the cohort. The leading respiratory conditions were acute upper respiratory infections (18.4%), pulmonary tuberculosis (4.1%), and COPD (4.6%). Respiratory diseases were significantly more common in older patients and were associated with both gender and ethnicity, with the Uygur ethnic group showing the highest prevalence. The findings suggest that age, gender, ethnicity, and environmental factors, such as the region’s climate, play pivotal roles in the epidemiology of respiratory diseases. These results underscore the need for targeted public health interventions aimed at managing respiratory conditions, especially for vulnerable populations such as the elderly and ethnic minorities in Xinjiang, China.

## Introduction

Respiratory diseases represent a major global health burden, with millions of people affected by conditions such as acute upper respiratory infections (1–5). Respiratory diseases contribute significantly to morbidity and mortality, and their prevalence is influenced by factors such as air pollution, climate, age, and ethnic disparities (6–8). Despite growing awareness of these diseases, epidemiological data from less studied regions, such as Xinjiang, remains limited.

Xinjiang, home to diverse ethnic groups such as Uygur, Han, and Kyrgyz, has unique climatic and geographical characteristics that could influence disease prevalence (9–11). The Atushi region, part of the Kizilsu Kirgiz Autonomous Prefecture, which experiences a temperate continental climate with extreme seasonal variations and low rainfall. These environmental conditions, combined with a high ethnic diversity, present a complex landscape for the study of respiratory diseases. Previous research has indicated that climate and environmental factors are crucial factors of respiratory health (12–15). Additionally, the interaction between genetic factors, socio-economic status, and access to healthcare further complicates disease distribution (16–19).

While studies from other parts of China, such as large urban centers, have explored the epidemiology of respiratory diseases, data from regions like Xinjiang remains scarce. Moreover, ethnic and geographical differences in the prevalence of these diseases have not been adequately addressed in the literature. The aim of this study is to analyze the demographic and clinical profiles of patients visiting the emergency department (ED) of The People’s Hospital of Atushi, with a focus on the prevalence and distribution of respiratory diseases.

## Materials and methods

### Study design and data collection

Data for this cross-sectional study were extracted from the electronic medical record (EMR) system of the People’s Hospital of Atushi from January 2024 to December 2024. Atushi People’s Hospital, established in 1972, is a comprehensive secondary-level hospital providing medical, health, rehabilitation, prevention, teaching, and family planning services. The hospital serves as a primary healthcare facility for both the urban and rural populations of the region. According to the results of the Seventh National Population Census, the permanent resident population of Atushi was approximately 290,900 in 2020. The hospital has 285 beds and employs 476 staff members. Annually, it admits over 14,000 patients, performs more than 2,000 surgeries, and handles upward of 40,000 outpatient visits. Respiratory diseases have a relatively high prevalence in the Atushi region of Xinjiang, which is hypothesized to be related to both the climate and the ethnic composition. This study aims to investigate the epidemiological characteristics of emergency department patients in the Atushi region of Xinjiang, with a particular focus on respiratory diseases, including acute upper respiratory infections, pulmonary tuberculosis, and chronic obstructive pulmonary disease (COPD).

Inclusion criteria were patients aged 14 years or older who visited the ED during the study period. Exclusion criteria included patients with missing data, which were identified and removed from the dataset. This study has been approved by the Medical Ethics Committee of the People’s Hospital of Atushi (No. ATSRMYY-KY-2025001).

### Participant characteristics

The demographic and clinical characteristics of patients were recorded, including age, gender, ethnicity, occupation, time of visit, season of visit, disease classification, and vital signs. Data were collected using a structured, electronic data entry form integrated into the hospital’s EMR system. This instrument was developed by the People’s Hospital of Atushi, pilot-tested for clarity and accuracy, and refined accordingly. Regarding the time of visit, the 24 h day was divided into eight 3 h intervals. Vital signs measured included systolic blood pressure (SBP), diastolic blood pressure (DBP), heart rate, respiratory rate, temperature, and oxygen saturation (SpO2). Patients were diagnosed with specific diseases based on clinical examination, laboratory tests, and imaging studies.

The classification of patients according to severity was based on the “Guidelines for Triage of Emergency Patients (Draft)” issued by the National Health and Family Planning Commission of China (20). This classification system divides patients into four categories: Level I (Extreme Danger): Immediate and critical condition, Level II (Severe): Urgent and requires immediate intervention, Level III (Emergency): Serious but stable condition, and Level IV (Non-Urgent): Minor and stable condition.

### Statistical analysis

Descriptive statistics were used to summarize patient characteristics, including means, standard deviations (SD), and proportions for continuous and categorical variables, respectively. The Chi-square test (χ^2^) was performed to assess the association between categorical variables such as age, gender, and ethnicity with the presence of respiratory diseases (pulmonary tuberculosis, acute upper respiratory infection, and COPD. A *p*-value of < 0.05 was considered statistically significant. All statistical analyses were conducted using SPSS version 26.0 (IBM Corp., Released 2019, IBM SPSS Statistics for Windows, Armonk, NY, United States).

## Results

### Patient demographics and baseline characteristics

Information on 8,963 participants was collected from the EMRs, of which 126 were excluded. A total of 8,837 patients were included in this study, consisting of 4,327 males (49.0%) and 4,510 females (51.0%) (Table 1). The age distribution of the patients was as follows: 3,996 patients (45.2%) were aged 14–34 years, 2,945 (33.3%) were aged 35–59 years, 1,819 (20.6%) were aged 60–84 years, and 77 patients (0.9%) were aged ≥ 85 years. The majority of patients were from the Uygur ethnic group (82.5%), followed by Han (13.7%), and Kyrgyz (3.8%) ethnicities.

**TABLE 1:** Baseline characteristics of patients.

Participant characteristics	Mean ± SD/*n* (%)
**Age (years)**	
14–34	3,996 (45.2%)
35–59	2,945 (33.3%)
60–84	1,819 (20.6%)
≥ 85	77 (0.9%)
**Gender**	
Male	4,327 (49.0%)
Female	4,510 (51.0%)
**Ethnicity**	
Han	1,210 (13.7%)
Kyrgyz	339 (3.8%)
Uygur	7,288 (82.5%)
**Occupation**	
Farmer	6,130 (69.4%)
Freelancer	39 (0.4%)
Student	1,775 (20.1%)
Worker	407 (4.6%)
Government employee	436 (4.9%)
Nurse	28 (0.3%)
Doctor	17 (0.2%)
Teacher	5 (0.1%)
**Time of visit**	
00:00–03:00	831 (9.45%)
03:00–06:00	254 (2.9%)
06:00–09:00	183 (2.1%)
09:00–12:00	1,136 (12.9%)
12:00–15:00	1,724 (19.5%)
15:00–18:00	1,335 (15.1%)
18:00–21:00	1,719 (19.5%)
21:00–24:00	1,655 (18.7%)
**Diagnosis of disease type**	
Respiratory disease	3,111 (35.2%)
Cardiovascular disease	811 (9.2%)
Urinary system disease	465 (5.3%)
Endocrine system disease	290 (3.3%)
Digestive system diseases	1,812 (20.5%)
Nervous system disease	456 (5.2%)
Blood system disease	7 (0.1%)
Cancer	17 (0.2%)
Trauma	1,145 (13.0%)
Skin disease	275 (3.1%)
Poisoning	44 (0.5%)
Physicochemical factor	16 (0.2%)
Other	388 (4.4%)
**Diagnosis of disease**	
Pulmonary tuberculosis	358 (4.1%)
Acute upper respiratory infection	1,625 (18.4%)
COPD	404 (4.6%)
**Classification of emergency patients**	
I	14 (0.2%)
II	229 (2.6%)
III	3,618 (40.9%)
IV	4,976 (56.3%)
**The way to the hospital**	
Self-admission	8,533 (96.6%)
Ambulance to hospital	304 (3.4%)
**Season**	
Spring	2,291 (25.9%)
Summer	2,447 (27.7%)
Autumn	2,163 (24.5%)
Winter	1,936 (21.9%)
**Vital sign**	
SBP, mmHg	123.1 ± 20.7
DBP, mmHg	73.8 ± 14.3
Temperature	36.5 ± 0.7
Heart rate, bpm	82.3 ± 10.4
Respiratory rate, breaths/min	20.2 ± 2.6
SpO_2_, %	95.1 ± 6.3
Total patients	8,837 (100.0%)

COPD, chronic obstructive pulmonary disease; SBP, systolic blood pressure; SpO_2_, peripheral oxygen saturation.

The mean and standard deviation of vital signs at the patient’s arrival were as follows: systolic blood pressure (SBP) was 123.1 ± 20.7 mmHg, diastolic blood pressure (DBP) was 73.8 ± 14.3 mmHg, temperature was 36.5 ± 0.7°C, heart rate was 82.3 ± 10.4 bpm, respiratory rate was 20.2 ± 2.6 breaths/min, and oxygen saturation (SpO_2_) was 95.1 ± 6.3%. In terms of triage classification, 14 patients (0.2%) were classified as Level I (extremely critical), 229 patients (2.6%) as Level II (severe), 3,618 patients (40.9%) as Level III (emergency), and 4,976 patients (56.3%) as Level IV (non-urgent).

### Disease prevalence and distribution

Out of the 8,837 patients, 3,111 (35.2%) were diagnosed with respiratory diseases (Table 1). The three primary types of respiratory diseases included acute upper respiratory infection (*n* = 1,625; 18.4%), pulmonary tuberculosis (*n* = 358; 4.1%), and COPD (*n* = 404; 4.6%). Other common disease categories included digestive system diseases (*n* = 1,812; 20.5%), trauma (*n* = 1,145; 13.0%), and cardiovascular diseases (*n* = 811; 9.2%). The remaining diseases, including those of the urinary, endocrine, nervous, and other systems, accounted for a smaller proportion of the cases.

### Respiratory diseases

#### Overall respiratory diseases

The prevalence of respiratory diseases was significantly higher among older patients (*p* < 0.001) (Table 2). Specifically, the prevalence of respiratory diseases was 34.0% in patients aged 14–34 years, 27.0% in patients aged 35–59 years, 50.0% in those aged 60–84 years, and 59.7% in those aged ≥ 85 years. There was also a statistically significant gender difference, with a higher prevalence in females (37.2%) compared to males (33.1%) (*p* < 0.001). Ethnicity was another significant factor, with the Uygur ethnic group having the highest prevalence of respiratory diseases (34.6%), followed by the Kyrgyz group (40.4%) and the Han group (37.1%) (*p* = 0.031).

**TABLE 2:** The association between respiratory diseases and patient gender, age, and ethnicity.

Participant characteristics	Non-respiratory disease (*N* = 5,726)	Respiratory disease (*N* = 3,111)	*P*-value
Age (years)			< 0.001
14–34	2,636 (66.0%)	1,360 (34.0%)	–
35–59	2,149 (73.0%)	796 (27.0%)	–
60–84	910 (50.0%)	909 (50.0%)	–
≥ 85	31 (40.3%)	46 (59.7%)	–
Gender			< 0.001
Male	2,895 (66.9%)	1,432 (33.1%)	–
Female	2,831 (62.8%)	1,679 (37.2%)	–
Ethnicity			0.031
Han	761 (62.9%)	449 (37.1%)	–
Kyrgyz	202 (59.6%)	137 (40.4%)	–
Uygur	4,763 (65.4%)	2,525 (34.6%)	–

#### Acute upper respiratory infection

Among the 1,625 patients diagnosed with acute upper respiratory infection, the prevalence was notably higher in younger age groups (Table 3). Specifically, 26.8% of patients aged 14–34 years, 15.9% of patients aged 35–59 years, and 4.7% of patients aged 60–84 years had acute upper respiratory infections (*p* < 0.001). Female patients had a higher prevalence of acute upper respiratory infection (20.2%) compared to male patients (16.5%) (*p* < 0.001). Ethnically, Uygur patients had the highest proportion of acute upper respiratory infection (16.6%), followed by Han (28.7%) and Kyrgyz patients (19.8%) (*p* < 0.001).

**TABLE 3:** The association between acute upper respiratory infection and patient gender, age, and ethnicity.

Participant characteristics	Non-acute upper respiratory infection (*N* = 7,212)	Acute upper respiratory infection (*N* = 1,625)	*P*-value
Age (years)			< 0.001
14–34	2,926 (73.2%)	1,070 (26.8%)	–
35–59	2,478 (84.1%)	467 (15.9%)	–
60–84	1,734 (95.3%)	85 (4.7%)	–
≥ 85	77 (100.0%)	0 (0%)	–
Gender			< 0.001
Male	3,615 (83.5%)	712 (16.5%)	–
Female	3,600 (79.8%)	910 (20.2%)	–
Ethnicity			< 0.001
Han	863 (71.3%)	347 (28.7%)	–
Kyrgyz	272 (80.2%)	67 (19.8%)	–
Uygur	6,080 (83.4%)	1,208 (16.6%)	–

#### Pulmonary tuberculosis

A total of 358 patients were diagnosed with pulmonary tuberculosis (Table 4). The prevalence of pulmonary tuberculosis was significantly higher in older patients, with 16.8% of those aged 60–84 years and 24.7% of those aged ≥ 85 years diagnosed with the disease (*p* < 0.001). There were no significant gender differences in pulmonary tuberculosis diagnosis (*p* = 0.939). The Uygur ethnic group had the highest prevalence of pulmonary tuberculosis (4.6%), followed by the Kyrgyz group (5.6%) and the Han group (0.2%) (*p* < 0.001).

**TABLE 4:** The association between pulmonary tuberculosis and patient gender, age, and ethnicity.

Participant characteristics	Non-pulmonary tuberculosis (*N* = 8,479)	Pulmonary tuberculosis (*N* = 358)	*P*-value
Age (years)			< 0.001
14–34	3,992 (99.9%)	4 (0.1%)	–
35–59	2,916 (99.0%)	29 (1.0%)	–
60–84	1,513 (83.2%)	306 (16.8%)	–
≥ 85	58 (75.3%)	19 (24.7%)	–
Gender			0.939
Male	4,151 (95.9%)	176 (4.1%)	–
Female	4,328 (96.0%)	182 (4.0%)	–
Ethnicity			< 0.001
Han	1,208 (99.8%)	2 (0.2%)	–
Kyrgyz	320 (94.4%)	19 (5.6%)	–
Uygur	6,951 (95.4%)	337 (4.6%)	–

#### COPD

Chronic obstructive pulmonary disease was diagnosed in 404 patients, with the prevalence significantly increasing with age (Table 5). Among the patients aged 14–34 years, none were diagnosed with COPD, while 2.6% of patients aged 35–59 years, 17.0% of patients aged 60–84 years, and 23.4% of patients aged ≥ 85 years were diagnosed with COPD (*p* < 0.001). No significant gender differences were found in COPD prevalence (*p* = 0.192). Ethnically, the Uygur group had the highest prevalence of COPD (5.4%), followed by the Kyrgyz group (2.7%) and the Han group (0.2%) (*p* < 0.001).

**TABLE 5:** The association between chronic obstructive pulmonary disease (COPD) and patient gender, age, and ethnicity.

Participant characteristics	Non-COPD (*N* = 8,433)	COPD (*N* = 404)	*P*-value
Age (years)			< 0.001
14–34	3,996 (100.0%)	0 (0.0%)	–
35–59	2,868 (97.4%)	77 (2.6%)	–
60–84	1,510 (83.0%)	309 (17.0%)	–
≥ 85	59 (76.6%)	18 (23.4%)	–
Gender			0.192
Male	4,142 (95.7%)	185 (4.3%)	–
Female	4,291 (95.1%)	219 (4.9%)	–
Ethnicity			< 0.001
Han	1,208 (99.8%)	2 (0.2%)	–
Kyrgyz	330 (97.3%)	9 (2.7%)	–
Uygur	6,895 (94.6%)	393 (5.4%)	–

## Discussion

Our study identified a high prevalence of respiratory diseases, with 35.2% of patients presenting with respiratory conditions. The analysis of the demographic and clinical characteristics of emergency department patients further revealed significant associations between age, gender, and ethnicity with the prevalence of respiratory diseases. Older age, female gender, and Uygur ethnicity were found to be risk factors for respiratory diseases.

Although the prevalence of pulmonary tuberculosis in China has been decreasing in recent decades, the southern region of Xinjiang remains a high-endemic area for tuberculosis, with a prevalence significantly higher than the national average (21–24). Pulmonary tuberculosis has long been associated with poverty, overcrowded living conditions, and underdeveloped healthcare infrastructure, all of which may disproportionately affect older populations who have had prolonged exposure to risk factors (25–27). Ethnically, the Uygur population had the highest prevalence of pulmonary tuberculosis, which aligns with earlier studies suggesting that some Uygur population are at higher risk for pulmonary tuberculosis due to genetic factors and healthcare access (28–30).

Chronic obstructive pulmonary disease, with a prevalence of 4.6%, followed the expected age-related trend, as older adults are more susceptible to chronic lung diseases (31, 32). No gender differences were found in the prevalence of COPD. Interestingly, the Uygur ethnic group again had the highest prevalence of COPD. Both previous studies and this study indicate that the prevalence of COPD among the Uyghur population is significantly higher than in other regions of China (33–36). Analysis by Li et al. (36) shows that lifestyle factors such as kitchen ventilation, cooking, and smoking are significantly associated with COPD. Uyghurs may have a greater preference for eating grilled meat, and since grilling food releases harmful substances like formaldehyde, benzene, and acrolein, this may exacerbate respiratory symptoms, reduce lung function, and increase the risk of developing COPD (36).

This study has several strengths. First, it is one of the few studies focusing on the epidemiology of respiratory diseases in the southwest Xinjiang, thus providing valuable insights into the health burdens faced by a unique population. In addition, the large sample size and detailed demographic data contribute to the robustness of the findings, allowing for nuanced analysis of factors such as age, gender, and ethnicity in the distribution of respiratory diseases.

However, there are several limitations that must be acknowledged. The single-center design limits the generalizability of the findings to other regions of China. The absence of data on certain environmental and lifestyle factors—such as air quality, smoking rates, and nutrition—means that some important confounders could not be accounted for.

In conclusion, this study provides an important contribution to understanding the epidemiology of respiratory diseases in the Atushi region of Xinjiang. The high burden of respiratory diseases in the ED is not only a reflection of current disease prevalence but also a signal that many cases might be avoided or better managed outside the emergency setting. For instance, a significant number of patients with exacerbated COPD or pulmonary tuberculosis complications could potentially receive timely and effective interventions through enhanced primary care. The higher burden of respiratory diseases among the elderly and ethnic minorities, especially the Uygur population, calls for targeted healthcare interventions and preventive measures tailored to the unique needs of these groups. For the elderly, public health interventions should include tailored screening programs, regular preventive care (like vaccinations), and simplified, accessible services—such as mobile or home-based visits—to enable early detection and prompt treatment. For ethnic minorities, health communications must be adapted to their language and cultural context while ensuring accessible preventive services, including TB screening, asthma/COPD management, and immunizations.

## Data Availability

The raw data supporting the conclusions of this article will be made available by the authors, without undue reservation.
